# Supplementary material for: Efficient and bright warm-white electroluminescence from lead-free metal halides
Source: Nat Commun. 2021 Mar 3;12:1421. doi: 10.1038/s41467-021-21638-x (PMC7930043; doi:10.1038/s41467-021-21638-x)
Supplement: Supplementary file 1 — Supplementary Information [file 41467_2021_21638_MOESM1_ESM.pdf]

Supplementary Information

Efficient and bright warm-white electroluminescence from lead-free metal halides

Hong Chen[1]†, Lin Zhu[1]†, Chen Xue[2]†, Pinlei Liu[1], Xuerong Du[1], Kaichuan Wen[1], Hao Zhang[1], Lei Xu[1], Chensheng Xiang[3], Chen Lin[4], Minchao Qin[5], Jing Zhang[6], Tao Jiang[1], Chang Yi[1], Lu Cheng[1], Chenglong Zhang[1], Pinghui Yang[1], Meiling Niu[1], Wenjie Xu[1], Jingya Lai[1], Yu Cao[1,2], Jin Chang[1], He Tian[3], Yizheng Jin[4], Xinhui Lu[5], Lang Jiang[6], Nana Wang[1]*, Wei Huang[1,2]* and Jianpu Wang[1]*

[1]Key Laboratory of Flexible Electronics (KLOFE) & Institute of Advanced Materials (IAM), Nanjing Tech University (NanjingTech), 30 South Puzhu Road, Nanjing 211816, China
[2]Shaanxi Institute of Flexible Electronics (SIFE), Xi'an Institute of Biomedical Materials & Engineering (IBME), Northwestern Polytechnical University (NPU), 127 West Youyi Road, Xi'an 710072, China
[3]China Center of Electron Microscope, State Key Laboratory of Silicon Material, School of Material Science & Engineering, Zhejiang University, Hangzhou 310027, China
[4]Center for Chemistry of High-Performance and Novel Materials, State Key Laboratory of Silicon Materials, and Department of Chemistry, Zhejiang University, Hangzhou 310027, China
[5]Department of Physics, The Chinese University of Hong Kong, Shatin 999077, Hong Kong, China
[6]Beijing National Laboratory for Molecular Sciences, Key Laboratory of Organic Solids, Institute of Chemistry, Chinese Academy of Sciences, Beijing 100190, China

Corresponding authors: Prof. Nana Wang (iamnnwang@njtech.edu.cn); Prof. Wei Huang (iamwhuang@nwpu.edu.cn); Prof. Jianpu Wang (iamjpwang@njtech.edu.cn).
†These authors contributed equally to this work.

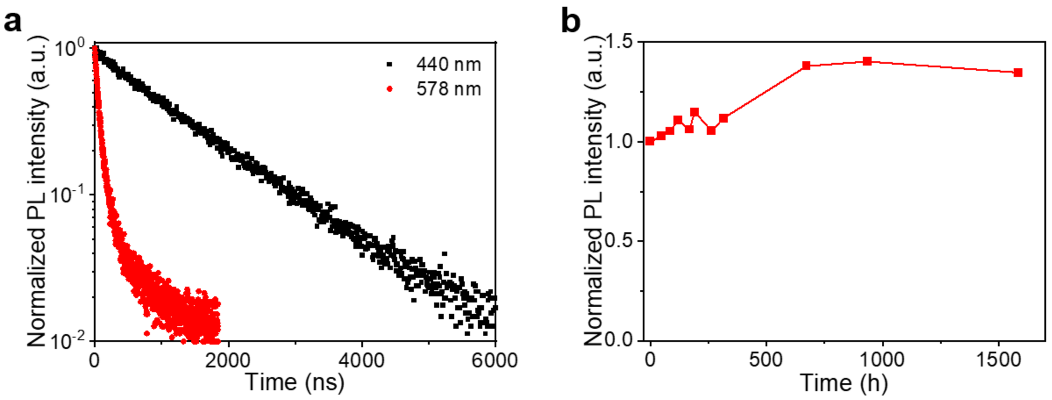

Supplementary Figure 1. Characterizations of cesium-copper-iodide films with Tween. a, Time-resolved PL decay curves. **b**, Evolution of PL intensity of the unencapsulated film stored in air with different time.

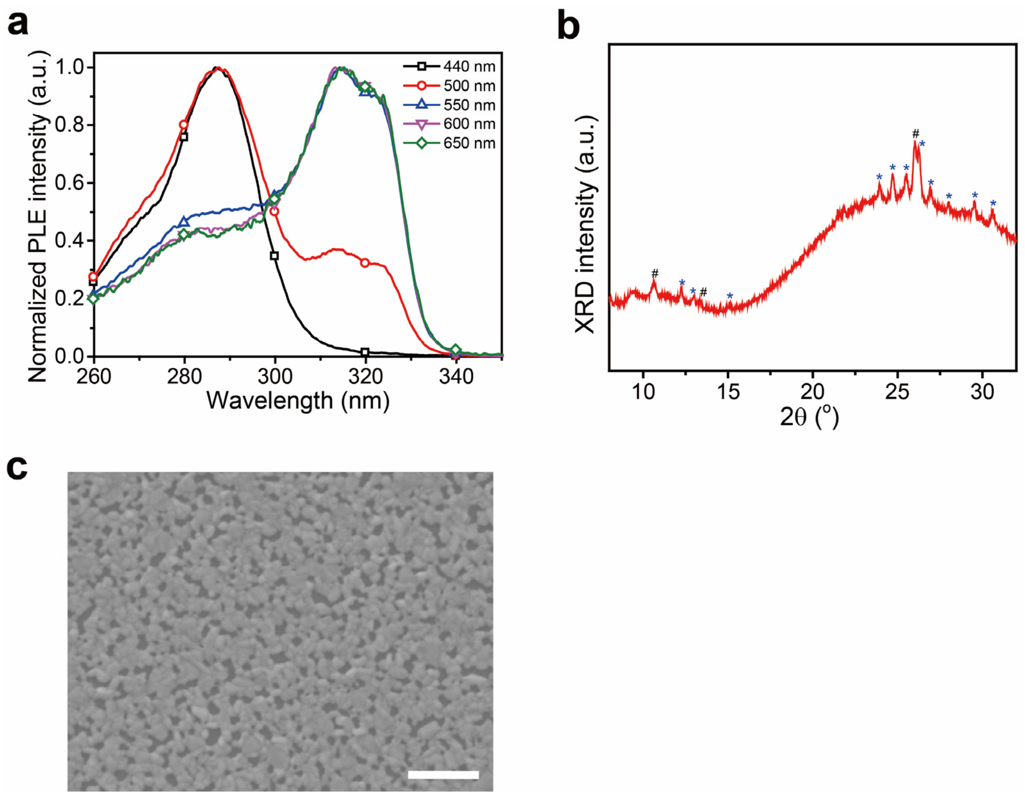

Supplementary Figure 2. Characterizations of cesium-copper-iodide films without Tween. a, Normalized PLE spectra at various emission wavelengths. **b**, XRD pattern. **c**, SEM image. Scale bar, 1 μm.

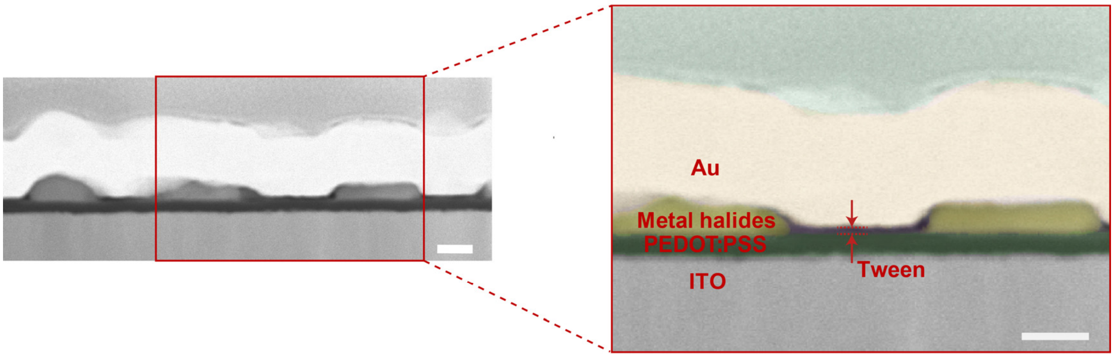

Supplementary Figure 3. Cross-sectional STEM image of the metal halide film (Scale bar, 100 nm). The STEM measurement was performed on a sample with structure of ITO/PEDOT:PSS/metal halides/Au. The layers in the right panel were tinted. Tween lies between the discrete cesium-copper-iodide crystallites, with a thickness of ~9 nm.

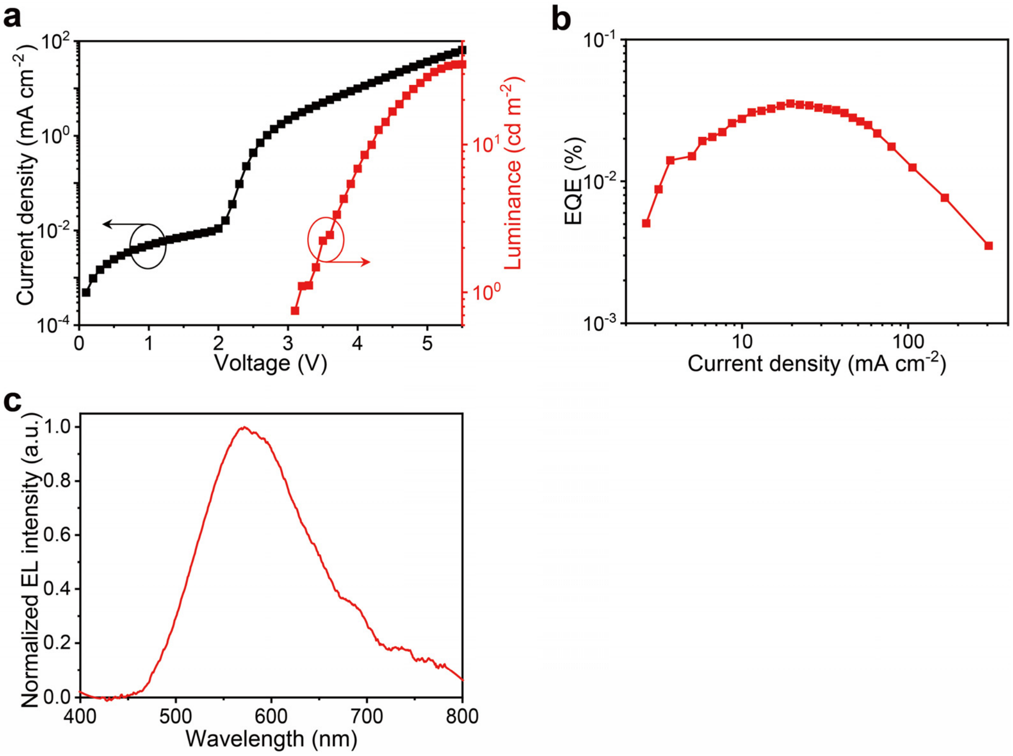

Supplementary Figure 4. Characterizations of devices fabricated from the cesium-copper-iodide films without Tween. a, Dependence of current density and luminance on the driving voltage. **b**, EQE versus current density. **c**, EL spectrum.

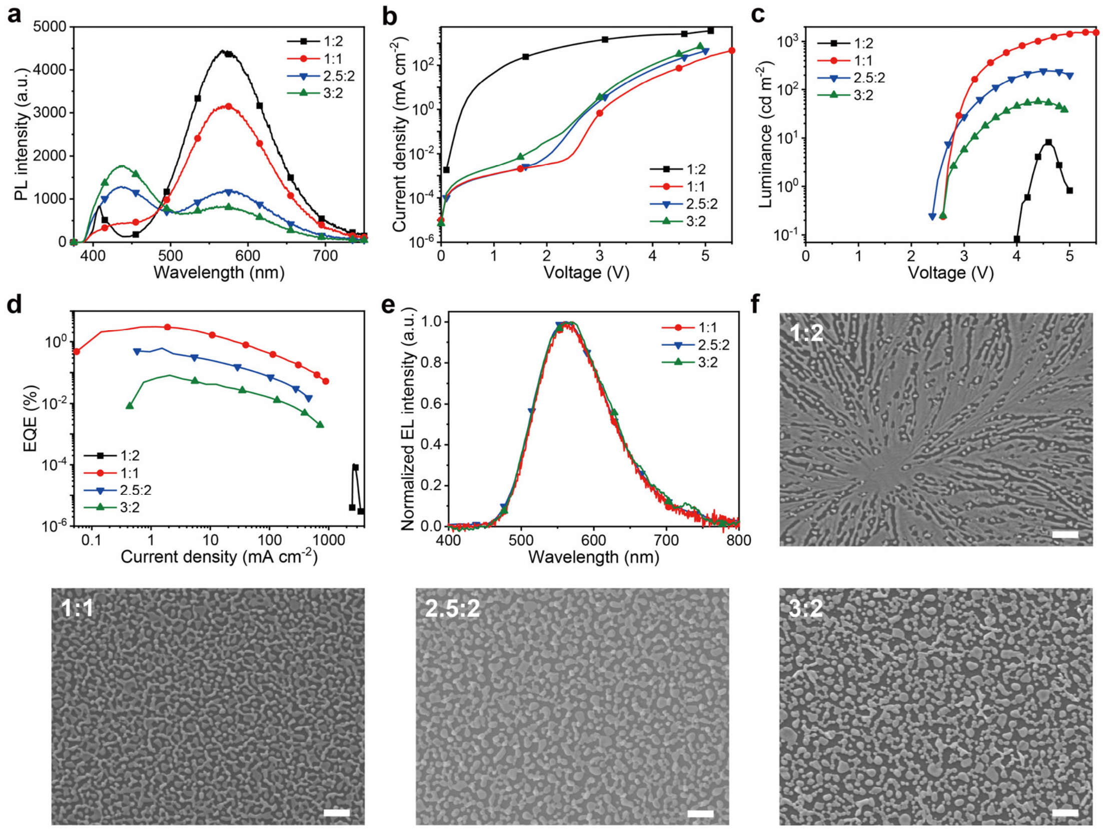

Supplementary Figure 5. Characterizations of films and devices fabricated from different ratios of CsI:CuI with Tween. a, PL spectra. **b**, Dependence of current density on the driving voltage. **c**, Dependence of luminance on the driving voltage. **d**, EQE versus current density. **e**, EL spectra. **f**, SEM images. Scale bar, 1 μm.

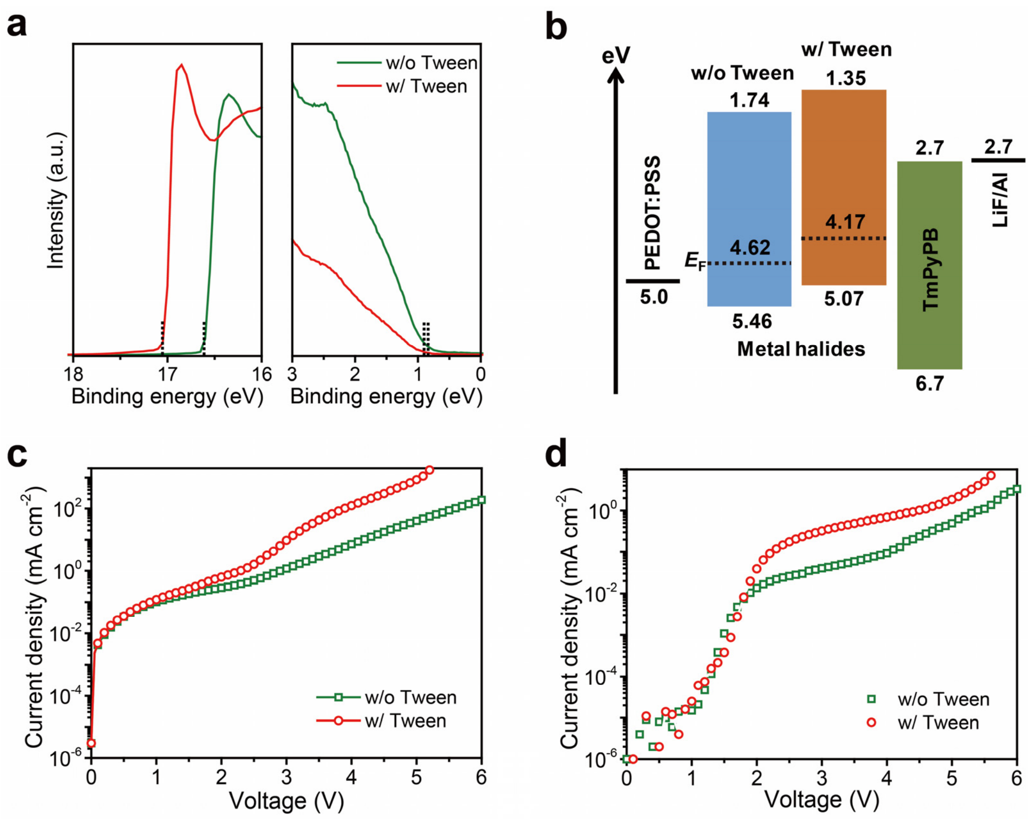

Supplementary Figure 6. Electronic properties of cesium-copper-iodide films. a, UPS spectra. After adding Tween, the valence band of cesium-copper-iodide film was changed from 5.46 to 5.07 eV. **b**, Schematic flat-band energy level diagram. The energy level values of cesium copper iodides were measured by UPS. **c**, J-V characterization of single-hole device with a structure of ITO/PEDOT:PSS/metal halide/TFB/MoO_3/Au. **d**, J-V characterization of single-electron device with a structure of ITO/SnO_2/PEIE/metal halide/TmPyPB/LiF/Al.

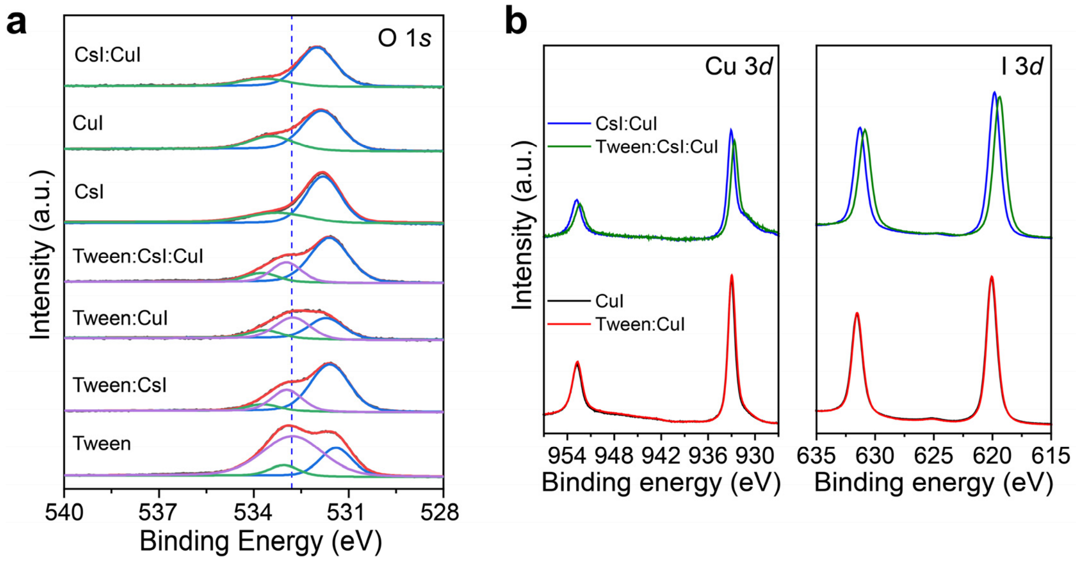

Supplementary Figure 7. XPS spectra. **a**, O 1*s* spectra. The O 1*s* lines were decomposed into two or three peaks. The red lines represent the raw data, while the blue, green lines represent the PEDOT:PSS and the purple lines represent the Tween. It shows that the peak of C=C-O group in PEDOT:PSS located at ~531.5 eV[1,2] shifts to 531.9 eV in the samples with CsI or CuI, which indicates that the PEDOT:PSS substrate has a strong coupling with CsI, CuI, and CsI:CuI films. But the Tween additive can reduce this interaction, which shows the corresponding C=C-O peak locating at ~531.5 eV. The peak at ~532.8 eV assigned to the oxygen of Tween has no shift after the inclusion of CuI, but shifts to higher binding energy in Tween:CsI sample. **b**, Cu 3*d* and I 3*d* spectra of CuI, Tween:CuI, CsI:CuI and Tween:CsI:CuI films.

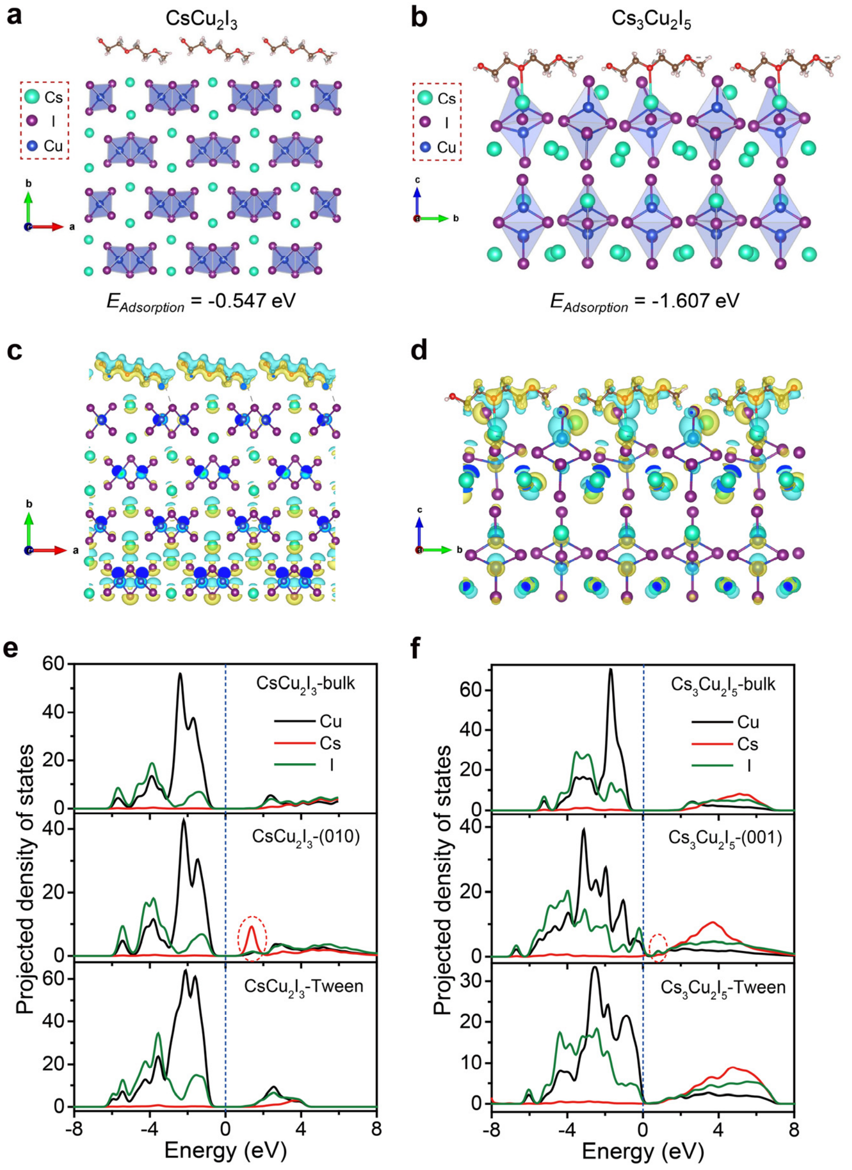

Supplementary Figure 8. Calculations of the interaction between cesium copper iodides and Tween. a-b, Optimized structures of $CsCu_2I_3$ (010)-additive (a) and $Cs_3Cu_2I_5$ (001)-additive (b), with listed binding energy between the free additive and the crystalline surfaces. **c-d**, Charge density differences ($\Delta\rho = \rho_{(crystal/additive)} - \rho_{crystal} - \rho_{additive}$) of the $CsCu_2I_3$ (010)-additive (c) and $Cs_3Cu_2I_5$ (001)-additive (d). The yellow and cyan regions indicate charge accumulation and charge depletion, respectively. **e-f**, Calculated projected density of states of the $CsCu_2I_3$ bulk, with Cs^+ exposed on the (010) facet and additive passivated $CsCu_2I_3$ (e) and those of the $Cs_3Cu_2I_5$ bulk, the (001) facet with Cs^+ exposure and the additive passivated $Cs_3Cu_2I_5$ (f). The Fermi levels are highlighted by the blue dash lines. The copper-based compounds with crystalline facets exposed with Cs^+ create a sub-gap state highlighted by the red circles.

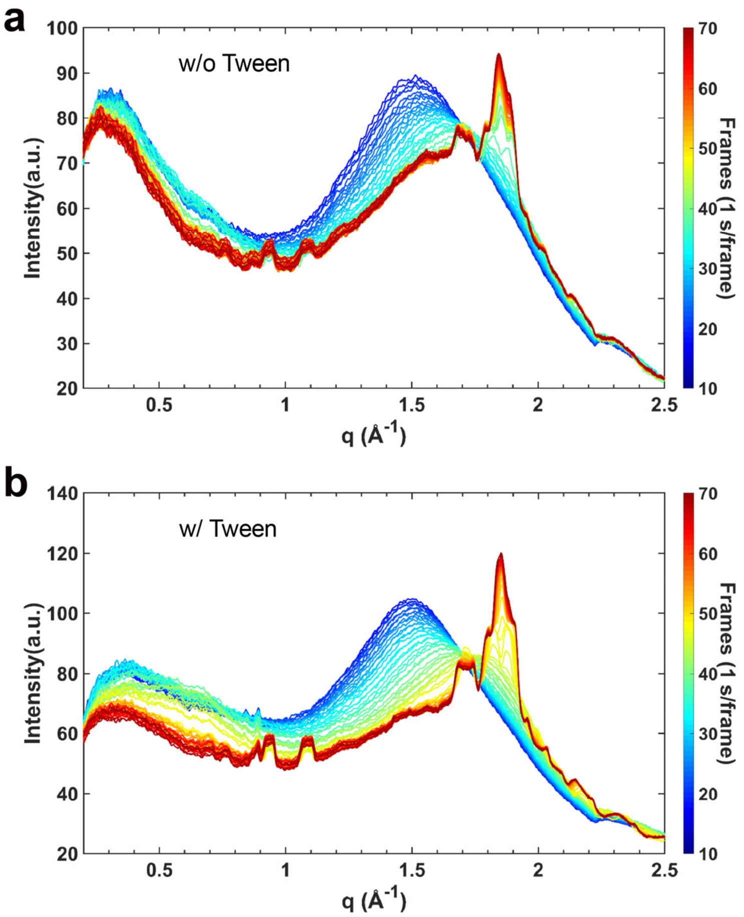

Supplementary Figure 9. Time-resolved GIWAXS profiles of cesium-copper-iodide films. a, Without Tween. **b**, With Tween.

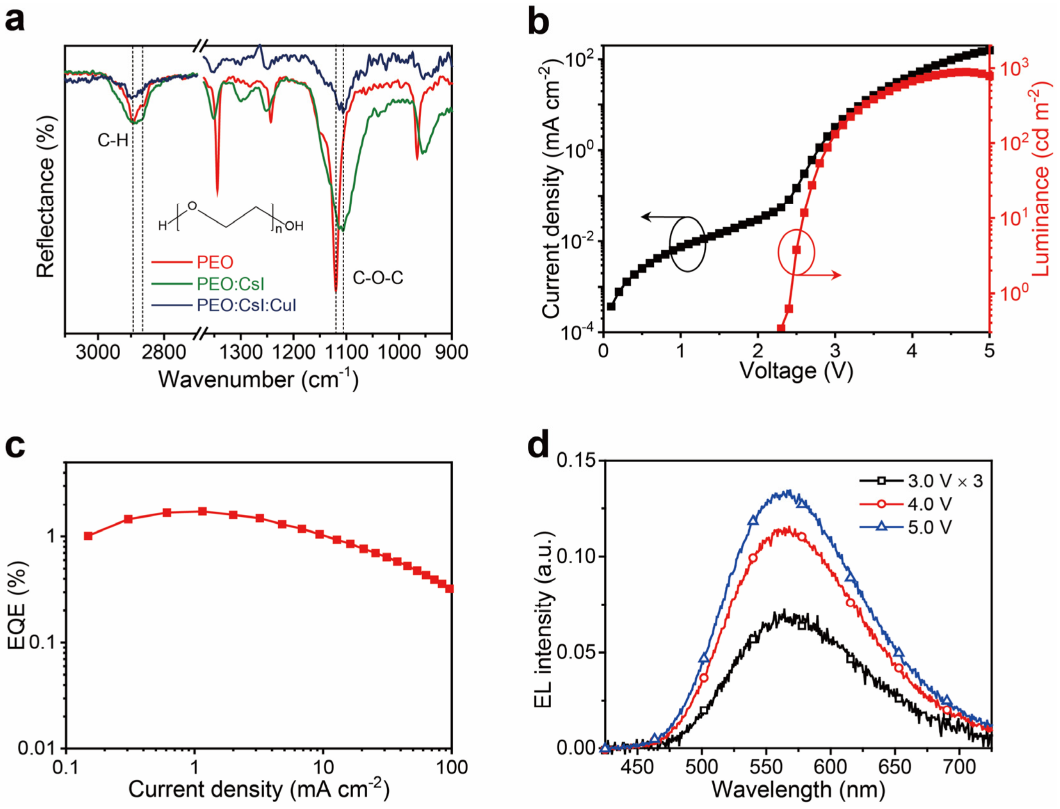

Supplementary Figure 10. Optoelectronic characteristics of the devices based on PEO. a, FTIR spectra. After adding PEO to CsI, the 1120 cm^{-1} peak ascribed to C-O-C stretching vibration of PEO is shifted to 1106 cm^{-1}, which is similar in the PEO:CsI:CuI sample. Inset, the structure of the PEO molecule. **b**, Dependence of current density and luminance on the driving voltage. **c**, EQE versus current density. The PEO-based devices reach a peak EQE of 1.7% and a maximum luminance of 890 cd m^{-2}, respectively. **d**, EL spectra at different voltages.

Supplementary Table 1. Comparison of our device with reported white metal halide LEDs.

Emitter	EL peak (nm)	Maximum luminance (cd m^{-2})	Peak EQE (%)	Reference
$CsPbBr_xCl_{3-x}$+MEH:PPV	470, 560	~150†	-	[3]
$PA_2CsPb_2I_7$+$CsPb(Br/Cl)_3$	493, 693	-	0.22	[4]
$MAPb(Br_{0.6}Cl_{0.4})_3$+organic compound	~500, 700	~14†	~0.001	[5]
$(C_6H_5C_2H_4NH_3)_2PbCl_2Br_2$	~475†	~70	-	[6]
$Cs_2Ag_{0.60}Na_{0.40}InCl_6$	~559†	~50†	-	[7]
$CsCu_2I_3$+mCP	~554	~14†	~0.15†	[8]
Tween-($Cs_3Cu_2I_5$:$CsCu_2I_3$)	565	1570	3.1	This work

†These data were estimated from the figures in refs [3,5–8].

Supplementary References:

1. Mengistie, D. A., Ibrahem, M. A., Wang, P.-C. & Chu, C.-W. Highly conductive PEDOT:PSS treated with formic acid for ITO-free polymer solar cells. *ACS Appl. Mater. Interfaces* **6**, 2292–2299 (2014).
2. Fan, Z., Li, P., Du, D. & Ouyang, J. Significantly enhanced thermoelectric properties of PEDOT:PSS films through sequential post-treatments with common acids and bases. *Adv. Energy Mater.* **7**, 1602116 (2017).
3. Yao, E.-P. *et al.* High-brightness blue and white LEDs based on inorganic perovskite nanocrystals and their composites. *Adv. Mater.* **29**, 1606859 (2017).
4. Mao, J. *et al.* All-perovskite emission architecture for white light-emitting diodes. *ACS Nano* **12**, 10486–10492 (2018).
5. Chang, C.-Y. *et al.* Perovskite white light-emitting diodes based on a molecular blend perovskite emissive layer. *J. Mater. Chem. C* **7**, 8634–8642 (2019).
6. Cai, P., Wang, X., Seo, H. J. & Yan, X. Bluish-white-light-emitting diodes based on two-dimensional lead halide perovskite $(C_6H_5C_2H_4NH_3)_2PbCl_2Br_2$. *Appl. Phys. Lett.* **112**, 153901 (2018).
7. Luo, J. *et al.* Efficient and stable emission of warm-white light from lead-free halide double perovskites. *Nature* **563**, 541–545 (2018).
8. Roccanova, R. *et al.* Bright luminescence from nontoxic $CsCu_2X_3$ (X = Cl, Br, I). *ACS Mater. Lett.* **1**, 459–465 (2019).
